# Tailoring the Stability of Ti-Doped Sr_2_Fe_1.4_Ti_x_Mo_0.6−x_O_6−δ_ Electrode Materials for Solid Oxide Fuel Cells

**DOI:** 10.3390/ma15228268

**Published:** 2022-11-21

**Authors:** Kun Zheng, Maciej Albrycht, Min Chen, Kezhen Qi, Paweł Czaja

**Affiliations:** 1 Department of Hydrogen Energy, Faculty of Energy and Fuels, AGH University of Science and Technology, al. A. Mickiewicza 30, 30-059 Krakow, Poland; 2 AGH Centre of Energy, AGH University of Science and Technology, ul. Czarnowiejska 36, 30-054 Krakow, Poland; 3 Decentralised Hydrogen–Maciej Albrycht, ul. Wały Dwernickiego 21/23a, lok. 8, 42-200 Częstochowa, Poland; 4 School of Materials Science and Energy Engineering, Foshan University, Foshan 528000, China; 5 College of Pharmacy, Dali University, Dali 671000, China; 6 Institute of Metallurgy and Materials Science, Polish Academy of Sciences, 25 Reymonta Str., 30-059 Krakow, Poland

**Keywords:** titanium doped Sr_2_(FeMo)O_6−δ_ perovskites, redox stability, anode materials, solid oxide fuel cells, crystal structure, thermal expansion, oxygen content, transport properties, compatibility with electrolytes

## Abstract

In this work, the stability of Sr_2_(FeMo)O_6−δ_-type perovskites was tailored by the substitution of Mo with Ti. Redox stable Sr_2_Fe_1.4_Ti_x_Mo_0.6−x_O_6−δ_ (x = 0.1, 0.2 and 0.3) perovskites were successfully obtained and evaluated as potential electrode materials for SOFCs. The crystal structure as a function of temperature, microstructure, redox stability, and thermal expansion properties in reducing and oxidizing atmospheres, oxygen content change, and transport properties in air and reducing conditions, as well as chemical stability and compatibility towards typical electrolytes have been systematically studied. All Sr_2_Fe_1.4_Ti_x_Mo_0.6−x_O_6−δ_ compounds exhibit a regular crystal structure with *Pm*-3*m* space group, showing excellent stability in oxidizing and reducing conditions. The increase of Ti-doping content in materials increases the thermal expansion coefficient (TEC), oxygen content change, and electrical conductivity in air, while it decreases the conductivity in reducing condition. All three materials are stable and compatible with studied electrolytes. Interestingly, redox stable Sr_2_Fe_1.4_Ti_0.1_Mo_0.5_O_6−δ_, possessing 1 μm grain size, low TEC (15.3 × 10^−6^ K^−1^), large oxygen content change of 0.72 mol·mol^−1^ between 30 and 900 °C, satisfactory conductivity of 4.1–7.3 S·cm^−1^ in 5% H_2_ at 600–800 °C, and good transport coefficients *D* and *k*, could be considered as a potential anode material for SOFCs, and are thus of great interest for further studies.

## 1. Introduction

Solid oxide fuel cells (SOFCs) with the advantage of high efficiency and fuel flexibility are among the most promising devices for the generation of electrical energy and heat from renewable and traditional energy sources, considerably reducing the emission of CO_2_ and other harmful gases (NO_x_, SO_x_, CO) [1,2,3,4,5] However, the high operational temperature (above 800 °C) of SOFCs leads to a high operational cost, limiting the choice of materials and delaying commercial applications of SOFCs. For practical use, SOFCs need to operate at much lower temperatures than the current range (≤800 °C) and still be able to generate a high-power production. Therefore, new anode [6,7,8] and cathode materials [9,10] with high stability and electrocatalytic activity are required to maintain a reasonable power output at temperature below 800 °C [11,12,13,14].

One group of the most interesting cathode and anode material candidates for SOFCs is the Sr_2_(FeMo)O_6_-type perovskite with Fe- and Mo-cations at B-site [15,16,17,18,19,20,21]. The B-site rock salt-type ordered Sr_2−x_Ba_x_M_1−y_Mo_y_O_6_ (M = Mg, Mn, Fe, Co, Ni) double perovskite-type compounds were initially proposed and evaluated as novel anode materials for SOFCs showing promising cell performance in different fuels, including hydrogen [22,23,24,25,26] and methane [27,28]. Generally, the choice of chemical composition in Sr_2_(Fe,Mo)O_6−δ_-type materials is governed by several crucial factors. The double perovskite (cations ordering) structure which favors the oxygen ion transport can be ensured by the considerable difference in oxidation state of B-site cations between Mo^6+^ or Mo^5+^ (in reducing atmospheres) and larger M^2+/3+^ cations (3*d* elements and Mg) [6,29]. The redox couple of M^2+^/M^3+^ and Mo^6+^/Mo^5+^ present in Sr_2_(Fe,Mo)O_6−δ_-type materials can not only favour an effective charge transport providing excellent conductivity [24,26], but also facilitate the creation of oxygen vacancies. For instance, Sr_2_FeMoO_6−δ_ oxide shows very high metallic conductivity with 1000 S cm^−1^ in reducing condition, while unfortunately the compound is not stable in air at high temperatures [26]. The modification of B-site cations in Sr_2_(Fe,Mo)O_6−δ_-type double perovskites can possibly bring good redox stability in both oxidizing and reducing conditions [30,31,32]. SrFe_0.75_Mo_0.25_O_3−δ_ [30,31], SrFe_0.5_Mn_0.25_Mo_0.25_O_3−δ_ [30], Sr_1−x_Ba_x_Fe_0.75_W_0.25_O_3−δ_ [33], and Sr_2_Fe_1.2_Mg_0.2_Mo_0.6_O_6−δ_ and Sr_2_Fe_0.9_Mg_0.4_Mo_0.7_O_6−δ_ perovskites [34] show good redox stability both in air and reducing conditions and have been studied as both cathode and anode materials for SOFCs. However, Sr_2_Fe_1.5_Mo_0.5_O_6−δ_ compound is sensitive to water, and the reaction of Sr_2_Fe_1.5_Mo_0.5_O_6−δ_ with H_2_O is a possible shortcoming of this perovskite as cathode and anode materials for SOFCs [35,36]. Mg-doped Sr_2_FeMo_2/3_Mg_1/3_O_6−δ_ double perovskite with a good tolerance to sulfur poisoning and carbon deposition was evaluated as a promising anode material candidate for SOFCs [37]. The Fe_B_-O-Fe_B′_ bonds in materials promote easy creation of oxygen vacancies and their fast migration. However, the low electrical conductivity (4–5 S cm^−1^ at 600–800 °C in air) may limit the application of such a material for SOFCs [37]. The copper-substituted Sr_2_Fe_1.5_Mo_0.3_Cu_0.2_O_6−δ_ material was investigated as a fuel electrode for the oxidation of H_2_ and CO_2_-CO reduction, showing improved reaction activity and durability, with an excellent SOFC power yield of 1.51 W cm^−2^ in H_2_ and a very good current density in the reduction of CO_2_ (with 1.94 A cm^−2^ at 1.4 V) [38]. The effect of Co-doping on the physicochemical and electrochemical properties of SrFe_0.45_Co_0.45_Mo_0.1_O_3−δ_ double perovskite has been investigated [39]. The material has been proposed as an air electrode for reversible solid oxide fuel cells, with high mobility of electron holes and oxygen ions. However, the recorded high thermal expansion coefficient is a limiting issue for the potential application of Co-doped oxide [39]. Sr_2_Mg_1−x_Co_x_MoO_6−δ_ perovskites with Co-doping at Mg-site, were investigated as novel anode materials for SOFCs, showing small anode polarization resistances [40], and the cobalt-doping positively contributes to the sinterability and ionic conductivity of materials [40]. However, the relatively weak bonding between Co-O is ascribed to the instability issue in anode condition, causing the reduction of Co to metallic cobalt. In addition, (PrBa)_0.95_(Fe_0.9_Mo_0.1_)_2_O_5+δ_ double perovskite with very high conductivity (59.2 S cm^−1^ in 5% H_2_, and 217 S cm^−1^ in air at 800 °C) was evaluated as an anode candidate for SOFCs, presenting excellent cell performance with 1.18 W cm^−2^ at 800 °C in H_2_S-containing fuel [8,11]. Therefore, the further modification of Sr_2_(FeMo)O_6_-type perovskites will contribute to the development of novel cathode and anode materials with high performance for SOFCs.

SrTiO_3_-derived perovskites are also of great interest as novel electrode materials for SOFCs, characterized by high redox stability in oxidizing and reducing conditions, and by a low economic and environmental impact [41,42,43]. Perovskites with mixed Fe- and Ti- at B-sites can favour the oxygen reduction reaction for SOFCs [44]. Ti- and Mo-containing Sr_2_TiMoO_6−δ_ double perovskite oxide was studied as novel anode material for SOFC, and it demonstrates excellent stability, sulphur poisoning resistance, and coking tolerance, as well as a good power output of 275 mW cm^−2^ in H_2_S-containing syngas at 850 °C [45]. Ba_x_Sr_1−x_Ti_1−y_Mo_y_O_3_ materials were synthesised and investigated as potential catalysts for the oxidation of CO and methane reforming [46]. In addition, the electrical conductivity and sintering properties of Ti-containing La_0.5_Sr_1.5_Ti_1.5_M_0.5_O_6−δ_ (M = Fe, Co and Ni) double perovskites were evaluated in terms of their application as new anode material candidates for [47]. Sr_2_ScTi_1−x_Mo_x_O_6_ (x = 0.1 and 0.5) double perovskites were studied as both cathode and anode materials for symmetrical solid oxide fuel cells, exhibiting a good power output of 218 mW cm^−2^ at 800 °C in humidified CH_4_ [48]. Ti-containing Sr_2_TiNi_0.5_Mo_0.5_O_6−δ_ perovskite was also proposed and systematically evaluated as a new anode material for SOFCs, generating a power density of 335 mW cm^−2^ at 800 °C in humidified H_2_ [49].

It has been reported that the electrocatalytic activity In the series of Sr_2_Fe_2−x_Mo_x_O_6−δ_ double perovskites enhances with the increase of Mo-doping content, which contributes to the improved performance of SOFCs cells in different fuels (H_2_ and methanol) [32], and SOFC with Sr_2_Fe_1.4_Mo_0.6_O_6−δ_ anode material shows much better electrochemical performance than the SOFC cell with redox stable Sr_2_Fe_1.5_Mo_0.5_O_6−δ_ electrode [32]. Sr_2_Fe_1.4_Mo_0.6_O_6−δ_ double perovskite possess very high electrical conductivity with 330 S cm^−1^ at 500 °C, nonetheless unfortunately it decomposes in air above 500 °C [50]. Interestingly, the Ti-doping at Fe-site in Sr_2_Fe_1.4−x_Ti_x_Mo_0.6_O_6−δ_ double perovskites significantly improves the structural stability of materials, which were systematically investigated as new anode material candidates for SOFCs [50]. Sr_2_Fe_1.3_Ti_0.1_Mo_0.6_O_6−δ_ anode-based SOFC cell delivers a promising power output with >0.64 W cm^2^ in wet hydrogen at 900 °C [50]. However, such a material is still not stable in air at a high temperature range. Therefore, in this work, the stability of Sr_2_Fe_1.4_Ti_x_Mo_0.6−x_O_6−δ_ (x = 0.1, 0.2 and 0.3) perovskites was tailored by the substitution of Mo with Ti at B-site. Sr_2_Fe_1.4_Ti_x_Mo_0.6−x_O_6−δ_ materials with excellent stability in both reducing and oxidizing atmospheres have been obtained. The crystal structure as a function of temperature, microstructure, redox stability, and thermal expansion properties in reducing and oxidizing atmospheres, oxygen content change and transport properties in air and reducing condition, as well as the chemical stability and compatibility towards typical solid electrolytes have been evaluated for the studied materials in terms of their application as electrode material candidates for SOFCs.

## 2. Materials and Methods

The synthesis of Sr_2_Fe_1.4_Ti_x_Mo_0.6−x_O_6−δ_ (x = 0.1, 0.2 and 0.3) materials was conducted using the high temperature method (solid state reaction), with the calculated amounts of SrCO_3_, Fe_2_O_3_, TiO_2_, and MoO_3_ (all compounds with ≥ 99.9% purity) chemicals. All required chemicals after milling in the high efficiency planetary ball mill (Spex Sample-Prep 8000 M, Spex Sampleprep, Metuchen, UK) were pressed into pellets and fired in air for 10 h at 1200 °C. The crystal structure properties of all obtained materials were investigated by the XRD measurements within 10–110 deg. 2 Theta range using the Panalytical Empyrean diffractometer (CuKα radiation, Malvern, UK).

The chemical stability of Sr_2_Fe_1.4_Ti_x_Mo_0.6−x_O_6−δ_ compounds was studied by the reduction of oxides in 5 vol.% H_2_ in argon for 10 h at 1200 °C. The XRD data refinement was applied applying the Rietveld method using GSAS/EXPGUI software [51,52]. Scanning electron microscopy (SEM) studies of reduced powders were conducted using FEI Nova NanoSEM 200 apparatus. The high temperature XRD studies were performed for the in-situ oxidation in air of reduced Sr_2_Fe_1.4_Ti_x_Mo_0.6−x_O_6−δ_ (x = 0.1, 0.2 and 0.3) samples using Panalytical Empyrean apparatus with Anton Paar HTK 1200N oven-chamber. The in-situ oxidation of Sr_2_Fe_1.4_Ti_x_Mo_0.6−x_O_6−δ_ sinters was also investigated with the thermal expansion measurements in air from room temperature to 900 °C using the Linseis L75 Platinum Series dilatometer (Selb, Germany).

Thermogravimetric (TG) studies were carried out on the TA Instruments Q5000IR apparatus (New Castle, DE, USA) and STA PT1600 TG with differential scanning calorimetry (DSC) studies from 30 to 900 °C in different conditions (in air and in 5 vol.% H_2_/Argon) with the rate of 2°·min^−1^. The buoyancy effect was also taken into account. The electrical conductivity (σ) of Sr_2_Fe_1.4_Ti_x_Mo_0.6−x_O_6−δ_ samples was recorded to 900 °C in air and 5 vol.% H_2_ in argon by a four-probe DC technique, on the dense cuboid shape sinters. The porosity effect of the studied sinters was also considered [53]. The oxygen diffusion coefficient *D* and surface exchange constant *k* of Sr_2_Fe_1.4_Ti_0.1_Mo_0.5_O_6−δ_ compound were studied using the mass relaxation technique in TA Instruments Q5000 IR on a very thin-sheet shape sample [54,55]. The mass relaxation measurements were performed with a very fast oxygen partial pressure change between 0.21 atm and 0.01 atm. The determination of coefficients *D* and *k* was conducted in custom-made Matlab code, based on Crank’s mathematical solutions [56]. The chemical stability and compatibility studies of Sr_2_Fe_1.4_Ti_x_Mo_0.6−x_O_6−δ_ (x = 0.1, 0.2 and 0.3) materials towards typical electrolytes, such as CGO20–Ce_0.8_Gd_0.2_O_1.9_ and LSGM–La_0.8_Sr_0.2_Ga_0.8_Mg_0.2_O_3−d_ solid electrolytes, were investigated by examining the XRD data gathered for the respective oxide and solid electrolyte mixtures (with a 50:50 wt.%), fired in air for 10 h at 1200 °C.

## 3. Results and Discussion

### 3.1. Crystal Structure and Microstructure

**Figure 1:**
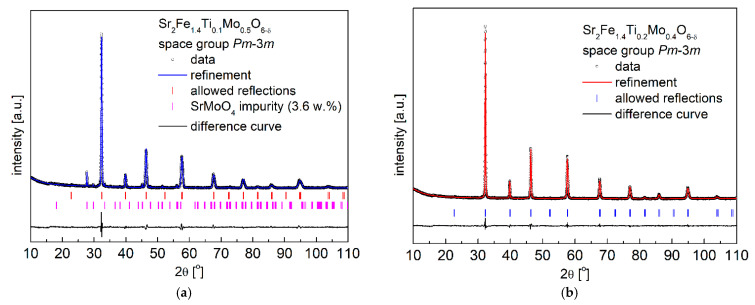

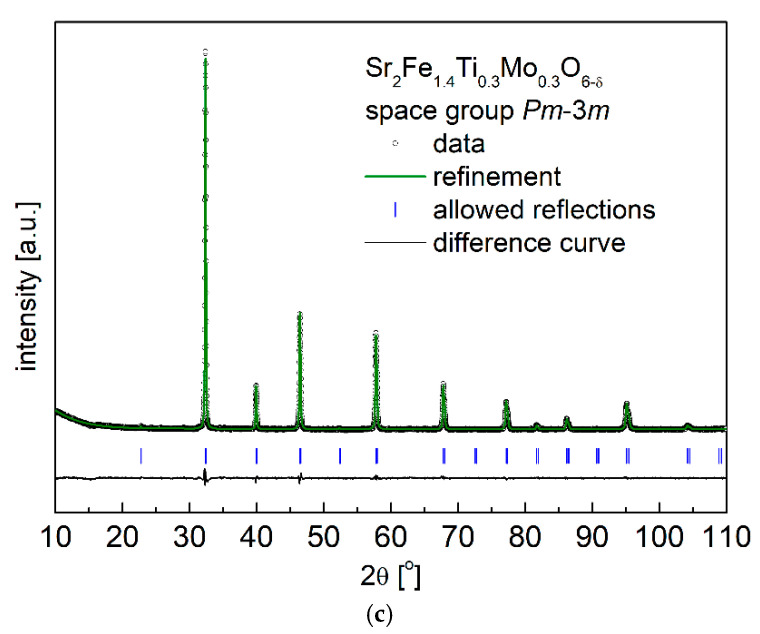
XRD patterns of the as-synthesized (**a**) Sr_2_Fe_1.4_Ti_0.1_Mo_0.5_O_6−δ_; (**b**) Sr_2_Fe_1.4_Ti_0.2_Mo_0.4_O_6−δ_ and (**c**) Sr_2_Fe_1.4_Ti_0.3_Mo_0.3_O_6−δ_ samples.

**Figure 2:**
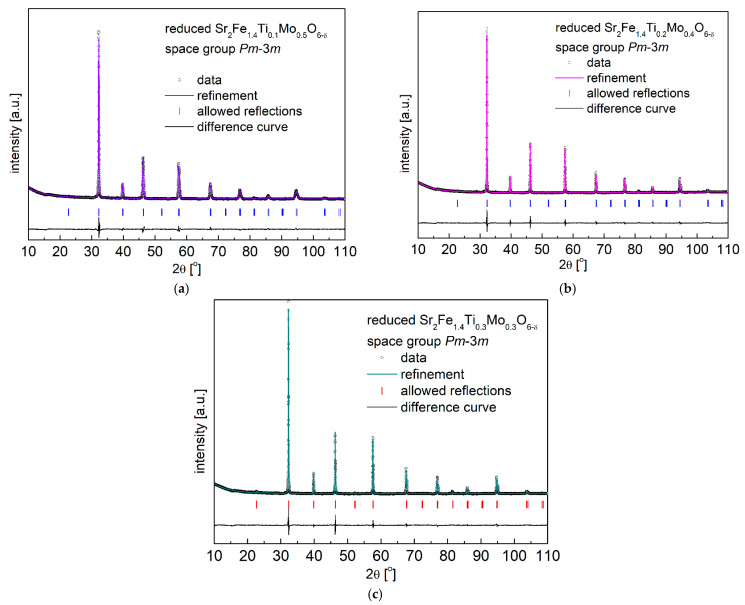
XRD patterns recorded for reduced (**a**) Sr_2_Fe_1.4_Ti_0.1_Mo_0.5_O_6−δ_; (**b**) Sr_2_Fe_1.4_Ti_0.2_Mo_0.4_O_6−δ_ and (**c**) Sr_2_Fe_1.4_Ti_0.3_Mo_0.3_O_6−δ_ oxides in 5 vol.% H_2_/Argon at 1200 °C for 10h.

The obtained XRD results with the Rietveld refinement in Figure 1 and Table 1 show that as-synthesized Sr_2_Fe_1.4_Ti_x_Mo_0.6−x_O_6−δ_ (x = 0.1, 0.2 and 0.3) samples exhibit simple perovskite crystal structure, belonging to the cubic *Pm*-3*m* space-group. However, the Sr_2_Fe_1.4_Ti_0.1_Mo_0.5_O_6−δ_ material synthesized in air possesses about 3.6% secondary phase (SrMoO_4_), which can be successfully removed by annealing the compound in reducing condition (see Figure 2a). As evidenced, the substitution of molybdenum by titanium in Sr_2_Fe_1.4_Ti_x_Mo_0.6−x_O_6−δ_ perovskites does not change the crystal symmetry but leads to a decrease of the unit cell parameter *a*. The doping of Ti^4+^ (with smaller oxidation state) at Mo^6+^ site contributes to the increase of Fe^4+^ (reducing the amount of Fe^3+^) and the content of oxygen vacancies in materials. The larger difference in ionic radius between Fe^3+^ (*r*_Fe3+_ = 0.645 Å) and Fe^4+^ (*r*_Fe4+_ = 0.585 Å) causes the decrease of the unit cell parameter, despite Ti^4+^ (*r*_Ti4+_ = 0.605 Å) presenting a slightly bigger ionic radius than Mo^6+^ (*r*_Mo6+_ = 0.59 Å). The geometric tolerance factor *t*_g_ of all studied materials was calculated using the following equation of $t_{g}=\frac{\left[A-O\right]}{\sqrt{2}\left[B-O\right]}$, where [A − O] and [B − O] are the refined geometric average values of interatomic distances of Sr-O and M-O (M: Fe, Ti and Mo), respectively [26]. The geometric tolerance factor was calculated to be *t*_g_ = 1.00 for all measured Sr_2_Fe_1.4_Ti_x_Mo_0.6−x_O_6−δ_ (x = 0.1, 0.2 and 0.3) samples, which indicates the presence of a regular crystal structure in the studied materials. Similar compositions, such as Sr_2_Fe_1.5_Mo_0.5_O_6−δ_ and SrFe_0.5_Mn_0.25_Mo_0.25_O_3−δ_ [30], Sr_2_TiFe_0.5_Mo_0.5_O_6−δ_ [57], and SrFe_0.45_Co_0.45_Mo_0.1_O_3−δ_ [39] oxides, also show a *Pm*-3*m* simple perovskite structure. Meanwhile, the Ti-doping at Fe-site in Sr_2_Fe_1.4−x_Ti_x_Mo_0.6_O_6−δ_ (x= 0 and 0.1) leads to a double perovskite structure with *Fm*-3*m* space group [50].

The redox stability of Sr_2_Fe_1.4_Ti_x_Mo_0.6−x_O_6−δ_ (x = 0.1, 0.2 and 0.3) materials was studied by annealing the compounds in 5 vol.% H_2_ in Argon at 1200 °C for 10h. The collected XRD data (Figure 2) after the reduction measurements show the reduced Sr_2_Fe_1.4_Ti_x_Mo_0.6−x_O_6−δ_ still possess the same crystal structure (*Pm*-3*m*), and the reduced B-site cations (Fe, Ti and Mo cations) with larger ionic radius contribute to a larger unit cell parameter *a* and volume *V* (see Table 1). Interestingly, among all investigated Sr_2_Fe_1.4_Ti_x_Mo_0.6−x_O_6−δ_ materials, Sr_2_Fe_1.4_Ti_0.1_Mo_0.5_O_6−δ_ oxide presents the smallest relative volume change ∆*V* = 0.52% between the reduced and oxidized samples. This indicates the possible application of such a compound in oxidizing and reducing conditions [54]. For comparison, the relative volume changes between the oxidized and reduced Sr_2_Fe_1.5_Mo_0.5_O_6−δ_ and Sr_2_Fe_0.9_Mg_0.4_Mo_0.7_O_6−δ_ oxides are larger and reach 1.18% [30] and 0.55% [34], respectively. The increase of Ti-doping in Sr_2_Fe_1.4_Ti_x_Mo_0.6−x_O_6−δ_ (x = 0.2 and 0.3) leads to a significantly large volume change ∆*V*, which can contribute to larger thermal expansion coefficients (TEC) in reducing and oxidizing atmospheres.

**Figure 3:**
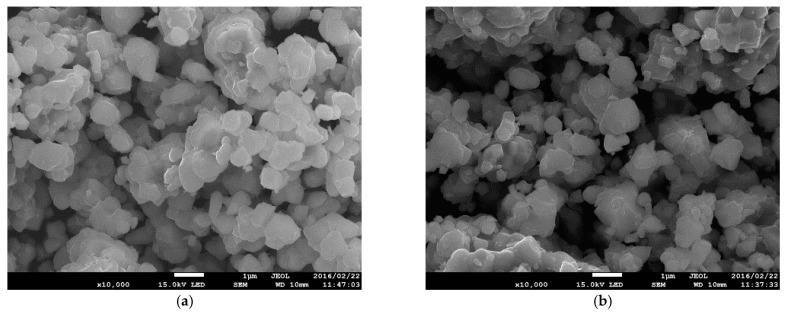

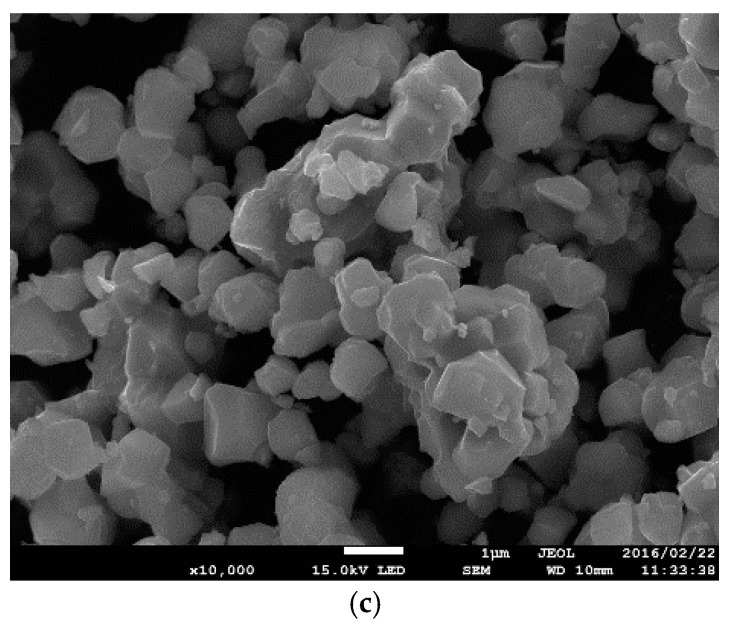
SEM micrographs of reduced (**a**) Sr_2_Fe_1.4_Ti_0.1_Mo_0.5_O_6−δ_; (**b**) Sr_2_Fe_1.4_Ti_0.2_Mo_0.4_O_6−δ_ and (**c**) Sr_2_Fe_1.4_Ti_0.3_Mo_0.3_O_6−δ_ powder.

SEM microphotographs of reduced Sr_2_Fe_1.4_Ti_x_Mo_0.6−x_O_6−δ_ (x = 0.1, 0.2 and 0.3) powders are shown in Figure 3. No substantial differences were observed in the microstructure for all studied materials, and the grain size is approximately 1 µm. Interestingly, the Ti-doping at Fe-site in Sr_2_Fe_1.4−x_Ti_x_Mo_0.6_O_6−δ_ materials (x = 0–0.2) introduces a totally different microstructure with well-sintered aggregates (primary grains/crystallites are not visible) reported in the work [50].

### 3.2. Redox Stability and Thermal Expansion Properties

**Figure 4:**
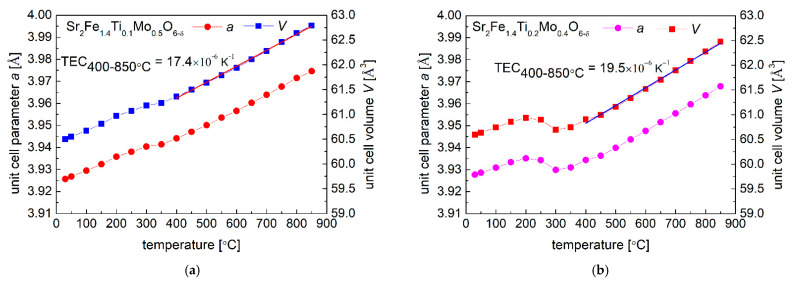

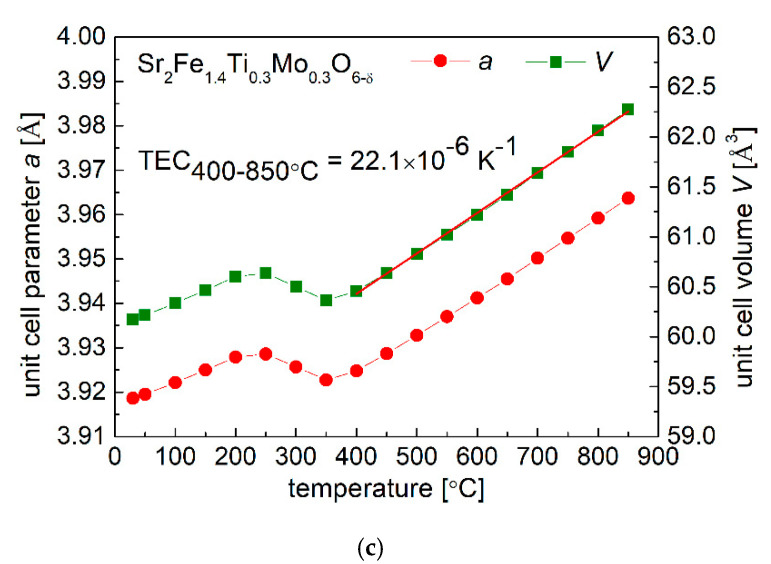
In-situ XRD measurements of oxidizing reduced (**a**) Sr_2_Fe_1.4_Ti_0.1_Mo_0.5_O_6−δ_; (**b**) Sr_2_Fe_1.4_Ti_0.2_Mo_0.4_O_6−δ_ and (**c**) Sr_2_Fe_1.4_Ti_0.3_Mo_0.3_O_6−δ_ materials.

**Figure 5:**
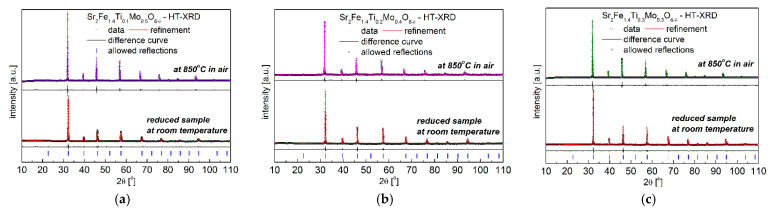
XRD patterns recorded at 30 °C and 850 °C in air for reduced (**a**) Sr_2_Fe_1.4_Ti_0.1_Mo_0.5_O_6−δ_; (**b**) Sr_2_Fe_1.4_Ti_0.2_Mo_0.4_O_6−δ_ and (**c**) Sr_2_Fe_1.4_Ti_0.3_Mo_0.3_O_6−δ_.

The in-situ oxidation of reduced Sr_2_Fe_1.4_Ti_x_Mo_0.6−x_O_6−δ_ (x = 0.1, 0.2 and 0.3) samples was examined by the high temperature XRD (HT-XRD) measurements in air. As can be observed in Figure 4, the oxidation of the reduced materials starts around 250 °C and finishes below 400 °C, accompanied by the significant change of unit cell parameter *a*. This is associated with the oxidation of reduced B-site cations (Fe, Ti, and Mo). The introduction of a higher content of titanium in Sr_2_Fe_1.4_Ti_x_Mo_0.6−x_O_6−δ_ (x = 0.2 and 0.3) leads to a more considerable unit cell parameter change. The initial linear increase of unit cell parameter recoded below 250 °C is attributed to the thermal expansion of reduced samples. Above 400 °C, the oxidized materials present a linear increase of unit cell parameter (indicating a linear thermal expansion behavior) to 850 °C in air, and the HT-XRD data allow to calculate the thermal expansion coefficient (TEC). The increase of Ti content in Sr_2_Fe_1.4_Ti_x_Mo_0.6−x_O_6−δ_ leads to an increase of TEC values. Sr_2_Fe_1.4_Ti_0.1_Mo_0.5_O_6−δ_ sample shows the lowest TEC among all studied materials, with TEC = 17.4 × 10^−6^ K^−1^. While Sr_2_Fe_1.4_Ti_0.3_Mo_0.3_O_6−δ_ presents a rather high TEC value of 22.1 × 10^−6^ K^−1^, which can be a shortcoming for applications as electrode materials for SOFCs. No phase transition was observed in the HT-XRD studies for all samples, and all Sr_2_Fe_1.4_Ti_x_Mo_0.6−x_O_6−δ_ materials are stable to 850 °C in air (Figure 5), possessing the same simple *Pm*-3*m* perovskite structure.

**Figure 6:**
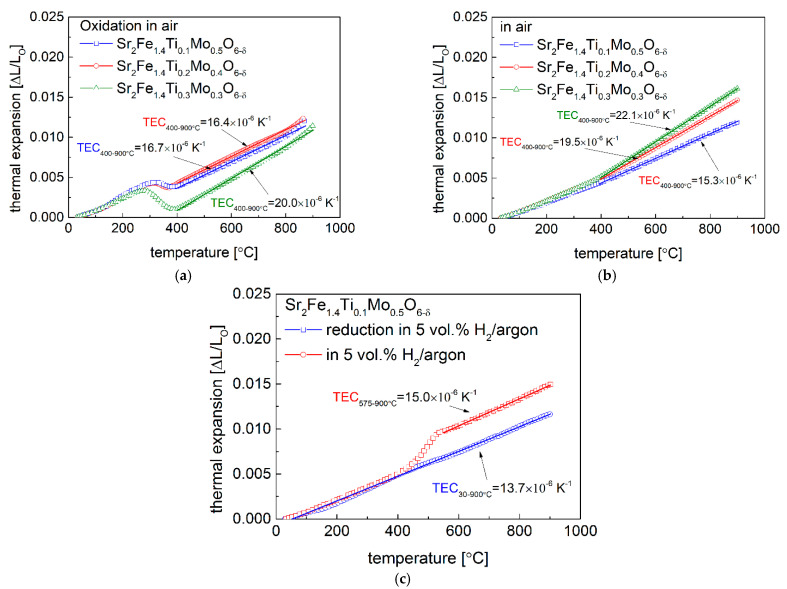
Thermal expansion behaviour of (**a**) oxidizing reduced Sr_2_Fe_1.4_Ti_x_Mo_0.6−x_O_6−δ_ sinters in air; (**b**) oxidized Sr_2_Fe_1.4_Ti_x_Mo_0.6−x_O_6−δ_ (x = 0.1, 0.2 and 0.3) samples in air; (**c**) reducing oxidized Sr_2_Fe_1.4_Ti_0.1_Mo_0.5_O_6−δ_ and the reduced sample in 5 vol. % H_2_/argon.

The in-situ oxidation of reduced Sr_2_Fe_1.4_Ti_x_Mo_0.6−x_O_6−δ_ (x = 0.1, 0.2 and 0.3) compounds was also investigated by dilatometry measurements in air (see Figure 6a). As in the case of HT-XRD measurements (Figure 4), the oxidation of reduced samples in dilatometry measurements (Figure 6a) occurs between 250 and 400 °C. A linear thermal expansion of reduced samples was recorded below 250 °C, and for oxidized materials a linear thermal expansion occurs in the range of 400–900 °C. Sr_2_Fe_1.4_Ti_0.1_Mo_0.5_O_6−δ_ sinter shows the smallest TEC with 16.4 × 10^−6^ K^−1^ among all studied samples, and the value is comparable with the TEC measured by HT-XRD studies (Figure 6a). Data presented in Figure 6a show that the TEC value of Sr_2_Fe_1.4_Ti_x_Mo_0.6−x_O_6−δ_ (x = 0.1, 0.2 and 0.3) increases with the increased content of Ti in materials.

**Figure 7:**
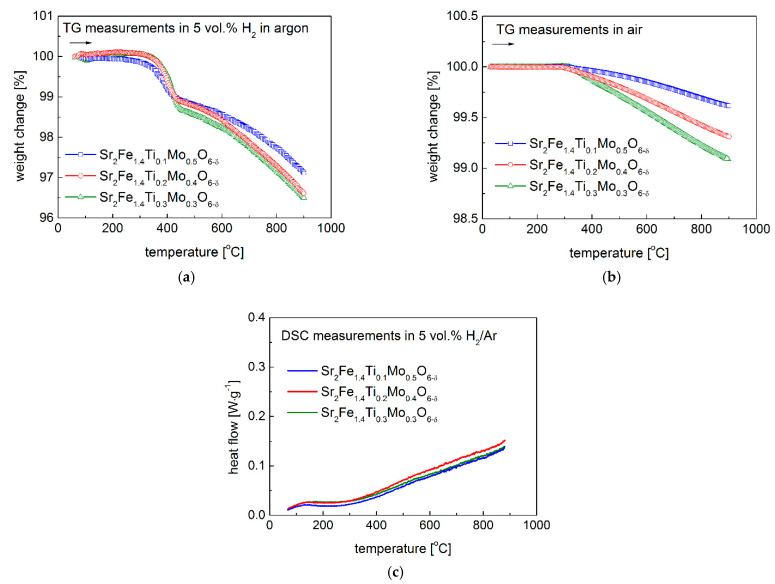
Oxygen content change of Sr_2_Fe_1.4_Ti_x_Mo_0.6−x_O_6−δ_ materials (**a**) in air and (**b**) in 5 vol.% H_2_ in argon; (**c**) results of DSC measurements for Sr_2_Fe_1.4_Ti_x_Mo_0.6−x_O_6−δ_ (x = 0.1, 0.2 and 0.3) oxides in 5 vol.% H_2_/Ar.

In addition, the thermal expansion behavior of oxidized Sr_2_Fe_1.4_Ti_x_Mo_0.6−x_O_6−δ_ (x = 0.1, 0.2 and 0.3) was studied in air (Figure 6b). For all three samples, two slopes with an obvious bending at 400 °C can be observed. The slope in the range of 400–900 °C, corresponding to a higher TEC, is related with the chemical expansion, caused by the reduction of B-site cations and loss of lattice oxygen, which was observed in the TG studies (Figure 7a). Sr_2_Fe_1.4_Ti_0.3_Mo_0.3_O_6−δ_ possesses the largest TEC value (22.1 × 10^−6^ K^−1^), while the TEC of Sr_2_Fe_1.4_Ti_0.1_Mo_0.5_O_6−δ_ is the smallest (15.3 × 10^−6^ K^−1^), which is close to the TEC values of typical solid electrolytes for SOFCs, such as: La_0.9_Sr_0.1_Ga_0.8_Mg_0.2_O_3−δ_ (TEC = 12.17 × 10^−6^ K^−1^), Zr_0.85_Y_0.15_O_2−δ_ (TEC = 10.8 × 10^−6^ K^−1^), and Ce_0.8_Gd_0.2_O_2−δ_ (TEC = 12.5 × 10^−6^ K^−1^) [26]. Moreover, the in-situ reduction of oxidized Sr_2_Fe_1.4_Ti_0.1_Mo_0.5_O_6−δ_ sample was conducted in 5 vol. % H_2_/argon (Figure 6c), and a considerable nonlinear change due to the reduction of B-site cations occurs above 400 °C, which corresponds well with the significant mass reduction of Sr_2_Fe_1.4_Ti_0.1_Mo_0.5_O_6−δ_ recorded in the TG measurement in Figure 7b. Meanwhile, a linear thermal expansion of reduced material was observed above 575 °C and it presents a relatively low TEC of 15.0 × 10^−6^ K^−1^. For the reduced Sr_2_Fe_1.4_Ti_0.1_Mo_0.5_O_6−δ_ sample in 5 vol. % H_2_/argon, it exhibits a linear thermal expansion, with a small TEC value of 13.7 × 10^−6^ K^−1^, which favours the application of such a material in reducing conditions. Therefore, in the case of the application of redox stable Sr_2_Fe_1.4_Ti_0.1_Mo_0.5_O_6−δ_ perovskite as electrode material for SOFCs, the TEC mismatch (causing delamination) problem is alleviated, possibly providing stable cell performance.

As can be observed in Table 2, the substitution of Mo by Ti at B-site in Sr_2_Fe_1.4_Ti_x_Mo_0.6−x_O_6−δ_ (x = 0.1, 0.2 and 0.3) materials leads to an increase of TEC values. Hence, the Ti-doping in Sr_2_Fe_1.4_Ti_x_Mo_0.6−x_O_6−δ_ does not benefit the thermal expansion properties and the titanium content in those materials should be restricted to a rather small level (such as x = 0.1).

### 3.3. Oxygen Content and Transport Properties

The oxygen content change of Sr_2_Fe_1.4_Ti_x_Mo_0.6−x_O_6−δ_ (x = 0.1, 0.2 and 0.3) was recorded by TG measurements in air and in 5 vol.% H_2_ in argon (see Figure 7a,b), respectively. For the TG studies in air, a significant mass loss of all three samples occurs above 300 °C, indicating the generation of additional oxygen vacancies in the studied perovskites, according to the following reaction: $O_{O}^{X}↔{1/2O}_{2}+V_{O}^{••}+{2e}^{-}$, which corresponds well with the chemical expansion of materials observed in Figure 6b. The substitution of Mo^6+^ by Ti^4+^ in the studied Sr_2_Fe_1.4_Ti_x_Mo_0.6−x_O_6−δ_ materials leads to the increase of oxygen vacancies. The data presented in Figure 7a indicate oxygen content decrease of ca. 0.1 mol·mol^−1^ for x = 0.1 sample up to 900 °C, ca. 0.17 mol·mol^−1^ for x = 0.2, and the largest change of ca. 0.20 mol·mol^−1^ for x = 0.3 material, respectively. In the reducing condition (Figure 7b), more oxygen vacancies were created in the materials, related with the reduction of B-site cations (Fe, Ti, Mo) to lower oxygen states (Fe^2+^/Fe^3+^, Ti^3+^ and Mo^5+^/Mo^4+^). The largest oxygen content change of ca. 0.86 mol·mol^−1^ was recorded for Sr_2_Fe_1.4_Ti_0.3_Mo_0.3_O_6−δ_ sample. In the case of Sr_2_Fe_1.4_Ti_0.2_Mo_0.4_O_6−δ_, the oxygen content decrease of ca. 0.84 mol·mol^−1^ was documented, while for Sr_2_Fe_1.4_Ti_0.1_Mo_0.5_O_6−δ_ it presents a relatively smaller change of 0.72 mol·mol^−1^.

In the supplementary DSC studies (Figure 7c), no endothermic or exothermic effects were observed for the investigated compounds, confirming no phase transition recorded, which was also documented in the in-situ HT-XRD studies (see Figure 4).

**Figure 8:**
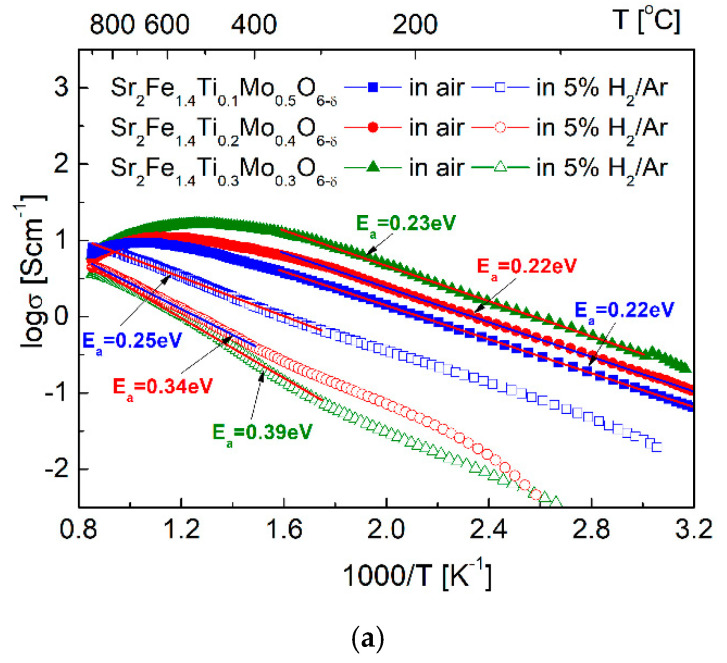

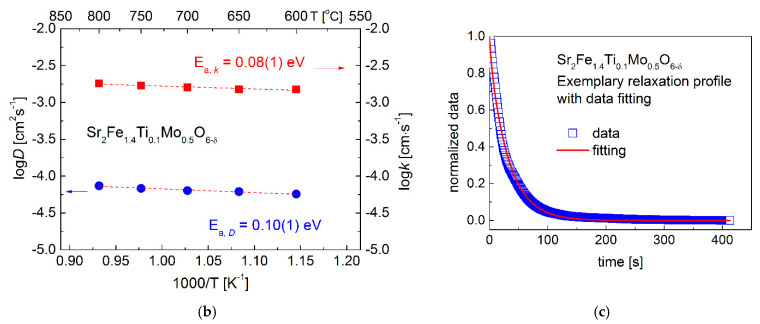
(**a**) Electrical conductivity of Sr_2_Fe_1.4_Ti_x_Mo_0.6−x_O_6−δ_ (x = 0.1, 0.2 and 0.3) oxides in air and 5 vol.% H_2_/Ar; (**b**) transport coefficients *D* and *k* determined as a function of temperature for Sr_2_Fe_1.4_Ti_0.1_Mo_0.5_O_6−δ_ sample; (**c**) an exemplary normalized relaxation profile with fitting for Sr_2_Fe_1.4_Ti_0.1_Mo_0.5_O_6−δ_ sinter.

The electrical conductivity σ data measured for Sr_2_Fe_1.4_Ti_x_Mo_0.6−x_O_6−δ_ (x = 0.1, 0.2 and 0.3) oxides in air (Figure 8a) show a maximum value of σ with the increase of temperature: it increases firstly and then decreases. All three materials initially exhibit a linear relationship below 400 °C with quite similar activation energy (0.22–0.23 eV), indicating small polaron conduction behavior. Similar behavior was also observed for Sr_2_Fe_1.2_Mg_0.2_Mo_0.6_O_6−δ_ and Sr_2_Fe_0.9_Mg_0.4_Mo_0.7_O_6−δ_ perovskites [34]. In Sr_2_Fe_1.4_Ti_x_Mo_0.6−x_O_6−δ_ materials, the electrons are transmitted via Fe^3+^-O^2^-/Fe^4+^ network in air. The Ti^4+^ substation of Mo^6+^ at B-site leads to the increase of Fe^4+^ content, thus favouring the electrical conductivities in air. Sr_2_Fe_1.4_Ti_0.3_Mo_0.3_O_6−δ_ sample shows the highest conductivity in air among all studied materials, with a peak value of 17.8 S·cm^−1^ at 500 °C. Meanwhile, in the case of Sr_2_Fe_1.4_Ti_0.2_Mo_0.4_O_6−δ_ compound, the maximum conductivity value of 11.5 S·cm^−1^ was observed at around 600 °C. For Sr_2_Fe_1.4_Ti_0.1_Mo_0.5_O_6−δ_, the maximum value (9.4 S·cm^−1^) was documented at 700 °C. The electrical conductivity decreases with a further increase of temperature, which is related with the release of oxygen from the lattice ($O_{O}^{X}↔{1/2O}_{2}+V_{O}^{••}+{2e}^{-}$) at high temperature range breaking the Fe^3+^-O^2—^Fe^4+^ network, causing the decrease of electrical conductivity. Similar behaviour was also observed in SrFeO_3_-based materials [21,30].

In the atmosphere of 5 vol.% H_2_/Ar, all Sr_2_Fe_1.4_Ti_x_Mo_0.6−x_O_6−δ_ (x = 0.1, 0.2, and 0.3) samples exhibit lower conductivity. In the reducing condition, the electrical conductivity of Fe-and Mo-containing perovskites is strongly related with the content of Fe^2+^/Fe^3+^–Mo^6+^/Mo^5+^ redox couples [24,26,30,38,50]. The Ti-doping in Sr_2_Fe_1.4_Ti_x_Mo_0.6−x_O_6−δ_ leads to the decrease of Fe^2+^/Fe^3+^–Mo^6+^/Mo^5+^ redox pairs, thus resulting in the decrease of electrical conductivity. Among all three investigated samples, Sr_2_Fe_1.4_Ti_0.3_Mo_0.3_O_6−δ_ oxide possesses the lowest conductivity (1.2–2.9 in 5% H_2_/Ar 600–800 °C) with the largest activation energy (*E*_a_ = 0.39 eV). In the case of Sr_2_Fe_1.4_Ti_0.1_Mo_0.5_O_6−δ_ perovskite, it shows relatively satisfactory conductivity value (4.1–7.3 S·cm^−1^ at 600–800 °C) with a small activation energy *E*_a_ = 0.25 eV. The measured electrical conductivity σ for Sr_2_Fe_1.4_Ti_0.1_Mo_0.5_O_6−δ_ is higher than the conductivity values (see Table 3) of Sr_2_Fe_1.5_Mo_0.3_Cu_0.2_O_6−δ_ [38], Sr_2_MgMoO_6−δ_ [58], Sr_2_Fe_0.9_Mg_0.4_Mo_0.7_O_6−δ_ [34], Sr_2−x_Ba_x_MgMoO_6−δ_, and Sr_2−x_Ba_x_MnMoO_6−δ_ [24,26], but smaller than the values of Sr_2_Fe_1.2_Mg_0.2_Mo_0.6_O_6−δ_ [34] and Sr_2_Fe_1.3_Ti_0.1_Mo_0.6_O_6−δ_ [50].

For Sr_2_Fe_1.4_Ti_0.1_Mo_0.5_O_6−δ_ sample, the chemical diffusion coefficient *D* and surface exchange constant *k* were evaluated by the mass relaxation study (see Figure 8b,c). The chemical diffusion coefficient *D* is in the range of 5.7 × 10^−5^ to 7.4 × 10^−5^ cm^2^ s^−1^ at 600–800 °C, with an activation energy *E*_a,D_ = 0.1 eV. While the surface exchange *k* is within the scope of 1.5 × 10^−3^–1.8 × 10^−3^ cm s^−1^, with a very small activation energy *E*_a,k_ = 0.08 eV. The determined chemical diffusion coefficient values are comparable with the *D* values measured for Sr_2_TiNi_0.5_Mo_0.5_O_6−δ_ [49], Sr_2_Fe_1.2_Mg_0.2_Mo_0.6_O_6−δ_ sample [34] and Sr_2_Fe_1.4_Mn_0.1_Mo_0.5_O_6−δ_ [59] materials. While the *k* values of Sr_2_Fe_1.4_Ti_0.1_Mo_0.5_O_6−δ_ are bigger than *k* values of Sr_2_Fe_1.2_Mg_0.2_Mo_0.6_O_6−δ_ sample [34] and Sr_2_Fe_1.4_Mn_0.1_Mo_0.5_O_6−δ_ [59]. The relatively good transport coefficients *D* and *k* evaluated for Sr_2_Fe_1.4_Ti_0.1_Mo_0.5_O_6−δ_ compound indicate good ionic transport properties in such a material.

**Table 3:** The crystal structure, electrical conductivity, thermal expansion coefficient (TEC), redox stability and compatibility with electrolytes, as well as the application of Sr_2_(Fe,Mo)O_6−δ_-based compounds.

Material	Structure	Electrical Conductivity [S·cm^−1^]	TEC Value [×10^−6^ K^−1^]	Stability	Towards Electrolyte	Application	Ref.
Sr_2_Fe_1.4_Ti_0.1_Mo_0.5_O_6−δ_	*Pm*-3*m*	9.4 at 700 °C in air; 4.1–7.3 in 5% H_2_ at 600–800 °C	15.3 in air	redox stable	stable with CGO and LSGM	cathode and anode candidate	this work
Sr_2_Fe_1.4_Ti_0.2_Mo_0.4_O_6−δ_	*Pm*-3*m*	11.5 at 600 °C in air; 1.6–3.5 in 5% H_2_ at 600–800 °C	19.5 in air	redox stable	stable with CGO and LSGM	cathode and anode candidate	this work
Sr_2_Fe_1.4_Ti_0.3_Mo_0.3_O_6−δ_	*Pm*-3*m*	17.8 at 500 °C in air; 1.2–2.9 in 5% H_2_ at 600–800 °C	22.1 in air	redox stable	stable with CGO and LSGM	cathode and anode candidate	this work
Sr_2_Fe_1.5_Mo_0.3_Cu_0.2_O_6−δ_	*Fm*-3*m*	0.06–0.36 in 5% H_2_ at 600–850 °C	-	decomposed in H_2_	-	fuel electrode	[38]
Sr_2_Fe_1.5_Mo_0.5_O_6−δ_	*Fm*-3*m or Pm*-3*m*	2.89–5.55 in 5% H_2_ at 600–850 °C; 13 in air at 400–600 °C	13.5–18.3 in air	redox stable	stable with CGO	cathode and anode candidate	[30,38]
Sr_2_MgMoO_6−δ_	*I*-1	0.8 in 5% H_2_ at 800 °C; 0.003 at 800 °C in air	-	stable in 5%H_2_	-	anode candidate	[58]
Sr_2−x_Ba_x_MnMoO_6−δ_	*P*2_1_/*n* and *Fm*-3*m*	0.24 to 1.41 in 5% H_2_	11.5 to 14.8 (x = 0) in air	stable in 5% H_2_/Ar	-	anode candidate	[24,26]
Sr_2−x_Ba_x_MgMoO_6−δ_	*I*4/*m* and *Fm*-3*m*	0.14 to 1.38 in 5% H_2_	13.8 to 18.2 (x = 0) in air	redox stable	-	anode candidate	[24,26]
Sr_2_Mg_0.95_Al_0.05_MoO_6−δ_	-	5.4 in 5% H_2_ at 800 °C	-	redox stable	Stable with LSGM and CGO, reacts with YSZ	anode candidate	[60]
SrFe_0.5_Mn_0.25_Mo_0.25_O_3−δ_	*Pm*-3*m*	3 at 850 °C in air; 10 at 850 °C in 5% H_2_	12.9 to 14.5 in air	redox stable	stable with CGO	cathode and anode candidate	[30]
Sr_2_Fe_1.2_Mg_0.2_Mo_0.6_O_6−δ_	*Fm*-3*m*	56.2 to 42.7 at 600–800 °C in air; 7.9 to 10.3 at 600–800 °C in 5% H_2_	14.6 to 16.7 in 5%H_2_; 12.9 to 14.6 in air	redox stable	stable with CGO, reacts with LSGM	cathode and anode candidate	[34]
Sr_2_Fe_0.9_Mg_0.4_Mo_0.7_O_6−δ_	*Fm*-3*m*	7.9 to 7.5 at 600–800 °C in air; 0.3 at 600–800 °C in 5% H_2_	14.2 to 15.1 in 5%H_2_; 13.5 to 15.7 in air	redox stable	stable with CGO, reacts with LSGM	cathode and anode candidate	[34]
Sr_2_Fe_1.5_Mo_0.4_Nb_0.1_O_6−δ_	*Pnma*	30 in air at 550 °C	16.1 in air	stable in air	stable with LSGM	cathode candidate	[61]
Sr_1.9_Fe_1.5_Mo_0.3_Cu_0.2_O_6−δ_	-	54.8 in air at 630 °C	19.4 in air	decomposed in H_2_	-	anode candidate	[62]
La_0.5_Sr_0.5_Fe_0.9_Mo_0.1_O_3−δ_	*Pm*-3*m*	2.7 to 6.7 at 600–800 °C in H_2_	15.1 in 5%H_2_; 13.4 in air	stable <750 °C in H_2_	stable with LSGM	cathode and anode candidate	[63]
Sr_2_FeMo_2/3_Mg_1/3_O_6−δ_	*Fm*-3*m*	4–5 in air at 600–800 °C; 9–13 in H_2_ at 600–800 °C	16.9 in air	redox stable	stable with LDC	anode candidate	[37]
Sr_2_FeMo_0.65_Ni_0.35_O_6−δ_	*I*4*/m*	55.4 in 5% H_2_ at 800 °C	-	decomposed in H_2_	stable with LDC	anode candidate	[18]
Sr_2_Fe_1.3_Ti_0.1_Mo_0.6_O_6−δ_	*Fm*-3*m*	220 to 160 at 500–800 °C in 5% H_2_	13.5 at 550 °C in air	stable in H_2_	stable with CGO	anode candidate	[50]
Sr_2_TiFe_0.5_Mo_0.5_O_6−δ_	*Pm*-3*m*	22.3 in H_2_ at 800 °C	11.2 in H_2_	stable in H_2_	stable with LSGM91 and CSO	anode candidate	[57]
Sr_2_TiNi_0.5_Mo_0.5_O_6−δ_	-	17.5 at 800 °C in hydrogen	12.8 in air	stable in H_2_	stable with LSGM	anode candidate	[49]
Sr_2−x_Ba_x_FeMoO_6−δ_	*I*4/*m* and *Fm*-3*m*	100 to 1000 in 5% H_2_	13.8 (for x = 0) in air	stable in 5%H_2_	stable with CGO	anode candidate	[24,26]
SrFe_0.45_Co_0.45_Mo_0.1_O_3−δ_	*Pm*-3*m*	298 at 300 °C in air	14.8 to 30.8 in air	stable in air	-	air electrode candidate	[39]
Sr_2_Mg_0.3_Co_0.7_MoO_6−δ_	*I*-1	9 to 7 at 600–800 °C in 5% H_2_	13.9 in air	-	-	anode candidate	[40]

CGO: Ce_0.8_Gd_0.2_O_1.9_, LSGM: La_0.8_Sr_0.2_Ga_0.8_Mg_0.2_O_3-d_, YSZ: Zr_0.92_Y_0.08_O_1.96_, LDC: La_0.4_Ce_0.6_O_2−d_, LSGM91: La_0.9_Sr_0.1_Ga_0.8_Mg_0.2_O_3−d_, CSO: Ce_0.8_Sm_0.2_O_1.9._

### 3.4. Chemical Stability and Compatibility with Electrolytes

**Figure 9:**
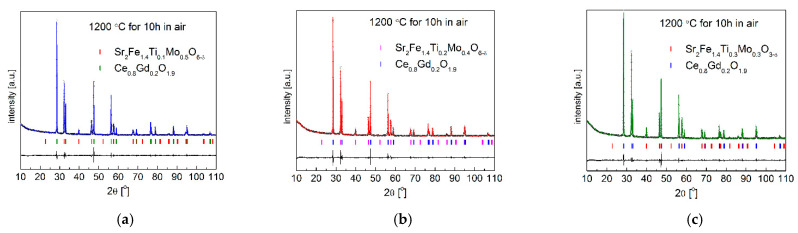
XRD patterns recorded for (**a**) Sr_2_Fe_1.4_Ti_0.1_Mo_0.5_O_6−δ_; (**b**) Sr_2_Fe_1.4_Ti_0.2_Mo_0.4_O_6−δ_ and (**c**) Sr_2_Fe_1.4_Ti_0.3_Mo_0.3_O_6−δ_ with Ce_0.8_Gd_0.2_O_1.9_ electrolyte, after firing in air at 1200 °C for 10 h.

**Figure 10:**
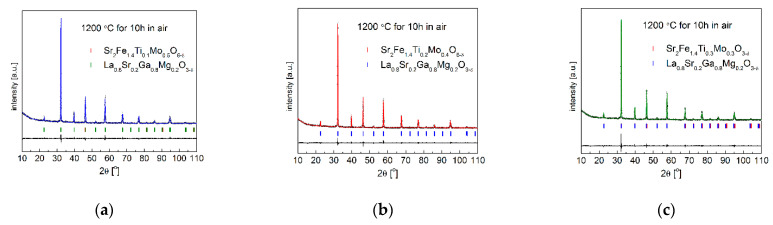
XRD patterns recorded for (**a**) Sr_2_Fe_1.4_Ti_0.1_Mo_0.5_O_6−δ_; (**b**) Sr_2_Fe_1.4_Ti_0.2_Mo_0.4_O_6−δ_ and (**c**) Sr_2_Fe_1.4_Ti_0.3_Mo_0.3_O_6−δ_ with La_0.8_Sr_0.2_Ga_0.8_Mg_0.2_O_3−δ_ electrolyte, after sintering at 1200 °C for 10 h in air.

The stability of cathode and anode oxides and their compatibility towards applied electrolytes at high temperature range are very critical for the electrode layer preparation (sintering) and a stable performance of cells. Therefore, the chemical stability of Sr_2_Fe_1.4_Ti_x_Mo_0.6−x_O_6−δ_ (x = 0.1, 0.2 and 0.3) oxides and their compatibility with typical solid electrolyte/buffer layer materials, such as Ce_0.8_Gd_0.2_O_1.9_–CGO20 and La_0.8_Sr_0.2_Ga_0.8_Mg_0.2_O_3−d_–LSGM, were evaluated by analyzing XRD data gathered for the respective materials and solid electrolyte powders (50:50 wt.%), which were fired in air for 10 h at 1200 °C. The Rietveld refined XRD patterns of Sr_2_Fe_1.4_Ti_x_Mo_0.6−x_O_6−δ_ with electrolyte (50:50 wt.%) powder mixtures are shown in Figure 9 and Figure 10. The XRD patterns present no chemical reaction occurring between the studied compounds and solid electrolytes (CGO20 and LSGM), confirming the good stability and compatibility of Sr_2_Fe_1.4_Ti_x_Mo_0.6−x_O_6−δ_ (x = 0.1, 0.2 and 0.3) materials with used electrolytes.

As presented in Table 4, the unit cell parameter of Ce_0.8_Gd_0.2_O_1.9_ from different mixtures is very similar, and the unit cell parameters of Sr_2_Fe_1.4_Ti_x_Mo_0.6−x_O_6−δ_ (x = 0.1, 0.2 and 0.3) from CGO20 mixtures are also very close to the values of appropriate materials recorded from LSGM mixtures. In addition, the unit cell parameter for LSGM from various mixtures is almost identical. This shows the good chemical stability of all investigated electrode materials and their compatibility towards electrolytes (Ce_0.8_Gd_0.2_O_1.9_ and La_0.8_Sr_0.2_Ga_0.8_Mg_0.2_O_3−d_). Interestingly, similar compositions Sr_2_Fe_1.2_Mg_0.2_Mo_0.6_O_6−δ_ and Sr_2_Fe_0.9_Mg_0.4_Mo_0.7_O_6−δ_ are compatible with Ce_0.8_Gd_0.2_O_1.9_ electrolyte but react with La_0.8_Sr_0.2_Ga_0.8_Mg_0.2_O_3−d_ [34]. Sr_2_Fe_1.3_Ti_0.1_Mo_0.6_O_6−δ_ (material unstable in air) is also compatible with Ce_0.8_Gd_0.2_O_1.9_ [50].

**Figure 11:**
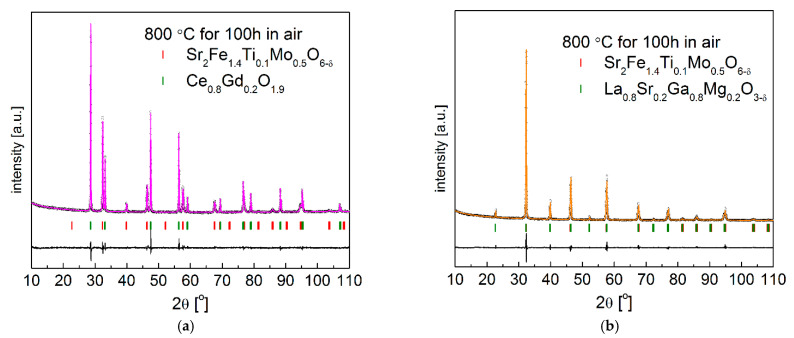
XRD patterns recorded for (**a**) Sr_2_Fe_1.4_Ti_0.1_Mo_0.5_O_6−δ_ with Ce_0.8_Gd_0.2_O_1.9_; (**b**) Sr_2_Fe_1.4_Ti_0.1_Mo_0.5_O_6−δ_ with La_0.8_Sr_0.2_Ga_0.8_Mg_0.2_O_3−δ_ electrolyte, after firing for 100h at 800 °C in air.

In addition, the long-term compatibility of Sr_2_Fe_1.4_Ti_0.1_Mo_0.5_O_6−δ_ towards Ce_0.8_Gd_0.2_O_1.9_ and with La_0.8_Sr_0.2_Ga_0.8_Mg_0.2_O_3−d_ solid electrolytes was evaluated, after firing for 100 h at 800 °C in air. As shown in Figure 11, Sr_2_Fe_1.4_Ti_0.1_Mo_0.5_O_6−δ_ sample has no reaction with Ce_0.8_Gd_0.2_O_1.9_ and La_0.8_Sr_0.2_Ga_0.8_Mg_0.2_O_3-d_ after annealing at 800 °C for 100 h. Moreover, the structural parameters gathered for Sr_2_Fe_1.4_Ti_0.1_Mo_0.5_O_6−δ_, Ce_0.8_Gd_0.2_O_1.9_ and La_0.8_Sr_0.2_Ga_0.8_Mg_0.2_O_3−d_ materials in Table 5 are very close to the respective results in Table 4, which indicate that the Sr_2_Fe_1.4_Ti_0.1_Mo_0.5_O_6−δ_ sample exhibits excellent stability and compatibility towards the used solid electrolytes, potentially providing a stable performance of Sr_2_Fe_1.4_Ti_0.1_Mo_0.5_O_6−δ_ in the cell.

## 4. Summary

In this work, the stability of Sr_2_(FeMo)O_6−δ_-type perovskites was successfully tailored by the substitution of Mo with Ti at B-site, and Sr_2_Fe_1.4_Ti_x_Mo_0.6−x_O_6−δ_ (x = 0.1, 0.2 and 0.3) perovskites with excellent redox stability in reducing and oxidizing conditions were obtained. All Sr_2_Fe_1.4_Ti_x_Mo_0.6−x_O_6−δ_ materials possess a regular simple perovskite structure with *Pm*-3*m* space group, showing excellent stability in both reducing and oxidizing conditions up to 1200 °C. All three materials present a similar microstructure with 1 μm grain size. The in-situ oxidation of reduced samples, observed by HT-XRD measurements and dilatometry studies, shows that the increased content of Ti doping at Mo-site in materials increases the TEC values. Sr_2_Fe_1.4_Ti_0.1_Mo_0.5_O_6−δ_ shows the lowest TEC with 15.3 × 10^−6^ K^−1^. In addition, Ti-doping also increases the oxygen content change and electrical conductivity in air, while it decreases the conductivity in reducing condition. Sr_2_Fe_1.4_Ti_0.3_Mo_0.3_O_6−δ_ sample presents the highest conductivity in air with 17.8 S·cm^−1^ at 500 °C, while the high TEC value of 22.1 × 10^−6^ K^−1^ can potentially limit the application. All three Sr_2_Fe_1.4_Ti_x_Mo_0.6−x_O_6−δ_ materials are stable and compatible with studied electrolytes (Ce_0.8_Gd_0.2_O_1.9_ and La_0.8_Sr_0.2_Ga_0.8_Mg_0.2_O_3−d_). 

Redox stable Sr_2_Fe_1.4_Ti_0.1_Mo_0.5_O_6−δ_ seems to be the most interesting among studied materials, with large oxygen content change of 0.72 mol·mol^−1^ between 30 and 900 °C, satisfactory conductivity of 4.1–7.3 S·cm^−1^ in 5% H_2_ at 600–800 °C, and good transport coefficients *D* and *k*, which indicate that such a material can be considered as a potential anode material for SOFCs and is of great interest for further studies.

## Figures and Tables

**Table 1:** Rietveld refinement results including unit cell parameters for the as-synthesized and reduced Sr_2_Fe_1.4_Ti_x_Mo_0.6−x_O_6−δ_ (x = 0.1, 0.2 and 0.3) oxides.

Composition	x = 0.1		x = 0.2		x = 0.3	
	* As Synthesized	Reduced	As Synthesized	Reduced	As Synthesized	Reduced
space group	*Pm*-3*m*	*Pm*-3*m*	*Pm*-3*m*	*Pm*-3*m*	*Pm*-3*m*	*Pm*-3*m*
*a* [Å]	3.9190 (1)	3.9257 (1)	3.9121 (1)	3.9277 (1)	3.9038 (1)	3.9186 (1)
*V* [Å^3^]	60.19 (1)	60.50 (1)	59.88 (1)	60.59 (1)	59.49 (1)	60.17 (1)
relative volume change ∆*V*	0.52%		1.19%		1.14%	
density [g/cm^3^]	5.55	5.52	5.51	5.45	5.48	5.42
CHI^2^	3.25	3.53	2.32	2.77	2.04	4.64
R_p_ (%)	1.38	1.65	1.26	1.46	1.15	1.53
R_wp_ (%)	1.99	2.39	1.71	2.14	1.61	2.51

* With around 3.6% SrMoO_4_ phase.

**Table 2:** Thermal expansion coefficients TEC [10^−6^ K^−1^] of Sr_2_Fe_1.4_Ti_x_Mo_0.6−x_O_6−δ_ (x = 0.1, 0.2 and 0.3) sinters from dilatometry studies and HT-XRD studies in air.

	HT-XRD (400–850 °C)	Dilatometer (400–900 °C, Oxidation in Air)	Dilatometer (400–900 °C, in Air for Oxidized Sinters)	Dilatometer (30–900 °C in 5 vol. % H_2_/Argon for Reduced Sinters)
Sr_2_Fe_1.4_Ti_0.1_Mo_0.5_O_6−δ_	17.4	16.4	15.3	-
Sr_2_Fe_1.4_Ti_0.2_Mo_0.4_O_6−δ_	19.5	16.7	19.5	13.7
Sr_2_Fe_1.4_Ti_0.3_Mo_0.3_O_6−δ_	22.1	20.0	22.1	-

**Table 4:** Structural parameters of Sr_2_Fe_1.4_Ti_x_Mo_0.6−x_O_6−δ_ (x = 0.1, 0.2 and 0.3) oxides with Ce_0.8_Gd_0.2_O_1.9_ and with La_0.8_Sr_0.2_Ga_0.8_Mg_0.2_O_3−d_ electrolytes from 50:50 wt.% mixtures fired in air for 10h at 1200 °C.

**Composition**	**x = 0.1**	**Ce_0.8_Gd_0.2_O_1.9_**	**x = 0.2**	**Ce_0.8_Gd_0.2_O_1.9_**	**x = 0.3**	**Ce_0.8_Gd_0.2_O_1.9_**
space group	*Pm*-3*m*	*Fm*-3*m*	*Pm*-3*m*	*Fm*-3*m*	*Pm*-3*m*	*Fm*-3*m*
*a* [Å]	3.9250 (1)	5.4263 (1)	3.9134 (1)	5.4259 (1)	3.9049 (1)	5.4256 (1)
*V* [Å^3^]	60.47 (1)	159.78 (1)	59.93 (1)	159.74 (1)	59.54 (1)	159.72 (1)
CHI^2^	3.29		4.35		3.08	
R_p_ (%)	1.85		1.95		1.76	
R_wp_ (%)	2.70		3.09		2.62	
**Composition**	**x = 0.1**	**La_0.8_Sr_0.2_Ga_0.8_Mg_0.2_O_3−δ_**	**x = 0.2**	**La_0.8_Sr_0.2_Ga_0.8_Mg_0.2_O_3−δ_**	**x = 0.3**	**La_0.8_Sr_0.2_Ga_0.8_Mg_0.2_O_3−δ_**
space group	*Pm*-3*m*	*Pm*-3*m*	*Pm*-3*m*	*Pm*-3*m*	*Pm*-3*m*	*Pm*-3*m*
*a* [Å]	3.9214 (1)	3.9140 (1)	3.9132 (1)	3.9132 (1)	3.9039 (1)	3.9142 (1)
*V* [Å^3^]	60.30 (1)	59.96 (1)	59.92 (1)	59.92 (1)	59.50 (1)	59.97 (1)
CHI^2^	2.03		1.98		2.23	
R_p_ (%)	1.66		1.66		1.70	
R_wp_ (%)	2.22		2.24		2.39	

**Table 5:** Structural parameters of Sr_2_Fe_1.4_Ti_0.1_Mo_0.5_O_6−δ_ sample with Ce_0.8_Gd_0.2_O_1.9_ and with La_0.8_Sr_0.2_Ga_0.8_Mg_0.2_O_3−d_ electrolytes after the long-term studies for 100 h at 800 °C in air.

Composition	Sr_2_Fe_1.4_Ti_0.1_Mo_0.5_O_6−δ_	Ce_0.8_Gd_0.2_O_1.9_	Sr_2_Fe_1.4_Ti_0.1_Mo_0.5_O_6−δ_	La_0.8_Sr_0.2_Ga_0.8_Mg_0.2_O_3−d_
space group	*Pm*-3*m*	*Fm*-3*m*	*Pm*-3*m*	*Pm*-3*m*
*a* [Å]	3.9268 (1)	5.4289 (1)	3.9229 (1)	3.9148 (1)
*V* [Å^3^]	60.55 (1)	160.00 (1)	60.37 (1)	60.00 (1)
CHI^2^	2.51		3.62	
R_p_ (%)	2.02		1.93	
R_wp_ (%)	2.89		2.82	

## Data Availability

The data presented in this study are available on request from the corresponding author.
